# From Plant Survival Under Severe Stress to Anti-Viral Human Defense – A Perspective That Calls for Common Efforts

**DOI:** 10.3389/fimmu.2021.673723

**Published:** 2021-06-15

**Authors:** Birgit Arnholdt-Schmitt, Gunasekaran Mohanapriya, Revuru Bharadwaj, Carlos Noceda, Elisete Santos Macedo, Ramalingam Sathishkumar, Kapuganti Jagadis Gupta, Debabrata Sircar, Sarma Rajeev Kumar, Shivani Srivastava, Alok Adholeya, KarineLeitão Lima Thiers, Shahid Aziz, Isabel Velada, Manuela Oliveira, Paulo Quaresma, Arvind Achra, Nidhi Gupta, Ashwani Kumar, José Hélio Costa

**Affiliations:** ^1^ Non-Institutional Competence Focus (NICFocus) ‘Functional Cell Reprogramming and Organism Plasticity’ (FunCROP), Coordinated from Foros de Vale de Figueira, Alentejo, Portugal; ^2^ Functional Genomics and Bioinformatics Group, Department of Biochemistry and Molecular Biology, Federal University of Ceará, Fortaleza, Brazil; ^3^ Plant Genetic Engineering Laboratory, Department of Biotechnology, Bharathiar University, Coimbatore, India; ^4^ Cell and Molecular Biotechnology of Plants (BIOCEMP)/Industrial Biotechnology and Bioproducts, Departamento de Ciencias de la Vida y de la Agricultura, Universidad de las Fuerzas Armadas-ESPE, Sangolquí, Ecuador; ^5^ National Institute of Plant Genome Research, Aruna Asaf Ali Marg, New Delhi, India; ^6^ Department of Biotechnology, Indian Institute of Technology, Roorkee, Uttarakhand, India; ^7^ Centre for Mycorrhizal Research, Sustainable Agriculture Division, The Energy and Resources Institute (TERI), TERI Gram, Gual Pahari, Gurugram, India; ^8^ MED—Mediterranean Institute for Agriculture, Environment and Development, Instituto de Investigação e Formação Avançada, Universidade de Évora, Évora, Portugal; ^9^ Department of Mathematics and CIMA - Center for Research on Mathematics and its Applications, Universidade de Évora, Évora, Portugal; ^10^ NOVA LINCS – Laboratory for Informatics and Computer Science, University of Évora, Évora, Portugal; ^11^ Department of Microbiology, Atal Bihari Vajpayee Institute of Medical Sciences & Dr Ram Manohar Lohia Hospital, New Delhi, India; ^12^ Hargovind Khorana Chair, Jayoti Vidyapeeth Womens University, Jaipur, India

**Keywords:** viral diseases, early cell reprogramming, ReprogVirus, somatic embryogenesis, alternative oxidase (AOX), aerobic fermentation, stress tolerance, SARS-CoV-2

## Abstract

Reprogramming of primary virus-infected cells is the critical step that turns viral attacks harmful to humans by initiating super-spreading at cell, organism and population levels. To develop early anti-viral therapies and proactive administration, it is important to understand the very first steps of this process. Plant somatic embryogenesis (SE) is the earliest and most studied model for *de novo* programming upon severe stress that, in contrast to virus attacks, promotes individual cell and organism survival. We argued that transcript level profiles of target genes established from *in vitro* SE induction as reference compared to virus-induced profiles can identify differential virus traits that link to harmful reprogramming. To validate this hypothesis, we selected a standard set of genes named ‘ReprogVirus’. This approach was recently applied and published. It resulted in identifying ‘CoV-MAC-TED’, a complex trait that is promising to support combating SARS-CoV-2-induced cell reprogramming in primary infected nose and mouth cells. In this perspective, we aim to explain the rationale of our scientific approach. We are highlighting relevant background knowledge on SE, emphasize the role of alternative oxidase in plant reprogramming and resilience as a learning tool for designing human virus-defense strategies and, present the list of selected genes. As an outlook, we announce wider data collection in a ‘ReprogVirus Platform’ to support anti-viral strategy design through common efforts.

## Background

Effective immunologic protection contributes to resilient behavior of higher organisms. It is essentially based on the diversity of innate and adaptive cell responses and cell memory tools (1–4). Immunologic responses are energy consuming and require efficient metabolic reprogramming. However, metabolic reorganization is only recently recognized as an integrated part of immunology (5–8). It is increasingly understood that plants and animals have similar responses and cell memory mechanisms to manage immunology (1, 3). These insights enable science to profit from experimental systems across organisms and to apply a higher degree of abstraction for gaining relevant knowledge on early reprogramming events that link to overall resilience.

### Somatic Embryogenesis (SE) – An Experimental Tool to Identify Markers for Early Reprogramming and Resilience

In plants, SE can be induced *in vitro* as a model for a resilient response upon severe stress of highly variable origins (9–18). SE induction depends essentially on the death of neighboring cells [(19); see also in (17)] and is defined as asexual regeneration of plants from single or few somatic cells, which can subsequently develop into an embryo in a similar process as it is known for zygotic embryogenesis in seeds [see reviews in (20)]. The discovery of SE in plants in 1958 revolutionized cell biology and stem cell research (9, 10). For the first time, it was revealed that totipotency could be acquired from differentiated somatic cells as it had been predicted by Haberlandt in 1902 (21, 22). SE is routinely used in plant biotechnology to massively propagate selected genotypes from individual plants. It can be utilized to help plants growing-out of virus threats, when propagation is induced from healthy parts of an infected plant (23). SE induction can be seen as an example of environment-inducible, molecular-physiological plasticity, a trait that is *per se* important marker for understanding resilient performance (17, 24–26).

It is common knowledge that energy-consuming reprogramming in eukaryotes is complex, individual- and context-dependent and integrates hormonal, epigenetic and metabolic actions regulated through a wide network of cell signaling factors, second messengers and transcription factors. Our group contributed to this knowledge with several research, perspective and reviewing papers [see e.g. in (11, 14, 26, 27)]. Typically, cell reprogramming covers dedifferentiation and *de novo* differentiation associated with autophagy and cell cycle regulation [see in (11, 17)]. Interaction within molecular networks relies upon cell origin, actual cell status, within cell distribution and structuration, cell communication and environmental signaling. Biochemical insights tell us that small variation at any level might have large consequences depending on thermodynamics, reactant and product concentrations, intermolecular forces, space organization and time. Consequently, relevant markers for reprogramming including those induced by viruses must be based on complex traits as confirmed by Costa et al. (Preprint 28).

Carbohydrate supply is essential for *in vitro* induction of SE (11, Preprint 28, Preprint 29). Sugars and sugar phosphates interact in plants and animals with hormone pathway networks and play central role in signaling to modulate energy metabolism and energy availability. Down-stream of sugars two important antagonistic protein kinases are involved in energy sensing and physiological adaptation (30–32). While sucrose non-fermenting-1-related protein kinase1 (SNRK1) is activated when energy is depleted (31, 33, 34), TOR (target of rapamycin, mTOR in mammals) is induced in situations of energy excess and stimulates cell cycle progression (G1/S and G2/M transitions) and cell proliferation (35). This stimulation involves transcription factors of the E2F family (36, 37). However, it was shown that a short six-hour pulse of one molar sucrose was sufficient to induce SE in hormone-free medium (16). This observation points to a more complex role of sucrose in cell reprogramming beyond energy supply. Sucrose is known to act as a signaling molecule (32, 38), in addition to acting as an osmotic stressor that can disrupt communication within and between cells (16).

Sucrose was also shown to trigger aerobic alcohol fermentation in support of respiration and synthesis of higher molecular weight compounds, such as, lipids (39). The phytohormone auxin and its distribution play critical roles for SE induction (40). However, sucrose could induce SE even in auxin-depleted medium (14). 2,4-dichlorophenoxyacetic acid (2,4-D), a synthetic herbicide that provides auxin activity, was shown to stimulate ethanol secretion in cultured carrot cells. Ethanol secretion was more dependent on sucrose availability than on oxygen availability, and linked to alcohol dehydrogenase (ADH) activity. Cell differentiation was shown to be critical for the amount of secreted ethanol (41, 42). Recently, Fan et al. (43) identified hormone and alcohol degradation pathways as the most activated during early stages of SE. Ethanol has been demonstrated to reduce ROS levels in stress performance and led to high induction of alternative oxidase (*AOX*) and glutathione-S-transferase transcripts relative to several other tested genes (44). Aerobic alcohol fermentation was found to play a critical role in controlling tissue level concentration of pyruvate in plants and thereby, adapt respiration rates primarily to energy status rather than to oxygen availability (45).

2,4-D is frequently used in plant biotechnology, because it can induce SE with high efficiency. It seems to impose higher oxidative stress levels than seen for native auxins (46, 47). Reactive oxygen species (ROS) enforced by ROS-induced ROS release (RIRR) and reactive nitrogen species (RNS) can integrate outer and inner cell signals and coordinate together adaptive cell and organism responses (48). Slight variations in ROS and RNS levels can have strong effects on cell fates (49, 50). Excess of nitric oxide (NO) and ROS can lead to production of peroxynitrite (ONOO^-^), which can cause nitration and subsequent inhibition of a broad range of cellular protein functioning and nitro-oxidative stress (51). ROS are known to interact with redox-sensitive protein cysteine thiol groups relevant for energy metabolism and metabolic channeling linked to cell differentiation and cell cycle regulation (51, 52, pre-print 53, 54). Downstream signaling pathways of NO constitute post-translational protein modifications by S-nitrosylation, including SUMOylation, phosphorylation, persulfidation and acetylation, which plays important role on altering protein functions either positively or negatively (55). Plant alcohol dehydrogenase 2 (ADH2) functions as nitroso-glutathione reductase (GSNOR) (56) and has high similarity to ADH5/GSNOR in human cells (Costa JH, not shown). GSNOR is involved in NO homeostasis and interferes with auxin signaling and polar auxin transport in higher plants (57). In animals, GSNOR was connected to mitochondria maintenance and cell longevity (58, 59). It can modulate redox signaling and, its overexpression in tomato could increase ROS and NO scavenging efficiency (60). Competence for SE induction was shown to be positively linked to the amount of anti-oxidant secondary plant compounds and enzymes (18, 26, 61–65). It is relevant to mention that high levels of NO can counteract SE induction, highly lightening the importance of balanced ROS/RNS homeodynamics in cells. Scavenging of NO by phytoglobins (66, 67) is suggested to integrate oxidative stress and auxin metabolism with the acquisition of SE competence. In plants, NO is produced mainly by the cytosolic nitrate reductase (NR) and mitochondrial electron transport-mediated nitrite to NO reduction (68).

### AOX Integrates ROS/RNS Signaling, Aerobic Fermentation and Respiration During Reprogramming - A Learning Tool for Virus Defense?

We hypothesized that a better understanding of the role of AOX during SE induction can help to reveal mechanisms that could be used to confront harmful virus-induced reprogramming in human cells. This hypothesis had been explored through original research (Preprint 28) and confirmed our approach.

AOX functions universally in a vast variety of organisms across all kingdoms (69). Most probably, *AOX* gene got transferred into eukaryotes from prokaryotes *via* primary endosymbiosis (70, 71). However, *AOX* is not present in vertebrates and arthropods and the majority of bacteria lost *AOX* during the course of evolution (72). Nevertheless, in 2005 an Alternative Consortium was created to explore a beneficial role of AOX in mitochondrial oxidative phosphorylation that could alleviate phenotypic effects of widespread OXPHOS deficiencies in human diseases (73, 74). Currently, *AOX* is being explored in animals, which overexpress *AOX* ubiquitously [e.g. (75)] as a tool to understand respiratory control mechanisms (76–78). Studies on transgenic *AOX*-mice revealed differential effects of AOX on acute and chronic hypoxia, which helped to better understand pulmonary oxygen sensing mechanisms vital e.g. for respiratory distress syndromes (79). Recently, it has been shown that viral infection, particularly respiratory viral infections upregulate ROS production [e.g. (80, 81)]. Overexpression of *AOX* in mouse displayed substantially reduced ROS generation (82). Also, cigarette smoke-induced mitochondrial stress and ROS production was shown to be relieved in *AOX*-mice attenuating lung dysfunction and tissue damage linked to chronic obstructive pulmonary disease (known as COPD) (83).

Mitochondrial AOX was proposed as functional marker for plant cell reprogramming (27). It demonstrated significant role in homeostasis, reprogramming and plant growth adaptation in response to diverse abiotic and biotic stresses (26, 84–90). Short- and long-term fine-tuning of *AOX* at transcriptional level was shown to be important for positive effects on performance (85, 91). Recently, relevance of *AOX* for predicting plant robustness from early reprogramming has been substantiated (26). In plants, virus tolerance is essentially regulated by salicylic acid, a hormone that acts on ROS accumulation (92). It involves a highly complex regulatory network, where AOX plays a role by modulating mitochondrial redox/ROS signaling (93). Fu et al. (94) revealed that NO acted as inducer of AOX in response to *Tobacco mosaic virus* (TMV) infection. *AOX* transcript accumulation took place when cytochrome-c-oxidase (COX) was inhibited by TMV, or NO or KCN.

In several applied plant systems of reproducibly stimulated morpho-physiological reprogramming, it was shown that early up- and down-regulation of *AOX* transcript levels is typical and coincides with critical phases of *de novo* induced morpho-physiologic events (induction, initiation, and realization). This included carrot SE induction and seed germination (24, 26), olive root induction for propagation from shoots (95, 96), callus induction from quiescent root tissue (97, 98), and *Hypericum perforatum* germination (99). In carrot seedlings, chilling also induced oscillating *AOX* transcript levels. *AOX* transcripts peaked after 45 minutes and prior to high induction of a specific anti-freezing gene only after 24h (98). These results are in agreement with state-of-the art knowledge on the importance of flexible short- and long-term fine-tuning of *AOX* at transcriptional level besides the protein level to enable known positive effects on plant performance (85, 91). To unravel the precise role of *AOX* and its isoforms during reprogramming integrated in complex signaling networks (100–102), it was suggested that measuring transient changes in respiration *in vivo* in seconds to minutes should be performed (103, 104).

The extraordinary role of AOX for reprogramming involves four major aspects for cell and tissue determination: (a) *AOX* is stress-induced and drives ROS level equilibration (105); *AOX* was shown to be involved in both scavenging and generation of NO (68). Cvetkovska and Vanlerberghe (106) demonstrated that overexpression of *AOX* led to lower NO production and *AOX* knockdown led to increasing NO. AOX scavenges electrons, thus it was expected to prevent in the mitochondrial electron transport chain electron leakage to nitrite and concomitant NO formation at the sites of complex III and complex IV. Later, Cvetkovska et al. (107) found that scavenging of NO could prevent NO inhibition of COX. Recently, Vishwakarma et al. (68) showed that bacterial elicitor flg22 treatment led to excess of NO, superoxide, peroxynitrite and tyrosine nitration. Moreover, *AOX* overexpression reduced peroxynitrite and tyrosine nitration suggesting that AOX-mediated NO removal can prevent downstream toxic products, (b) AOX is critical for mitochondrial ROS signal transduction towards mitochondria-nucleus retrograde communication (108–110), (c) AOX contributes to prevent excessive plant cell death by regulating ROS levels (17, 111, 112), and, (d) pyruvate is a major metabolic regulator of AOX (104, 113–117), which links to the role of sugar and the central branch point between respiration and fermentation (118). AOX activation can avoid energy and carbon shortage for anabolism by maintaining the tricarboxylic acid cycle active also when oxygen concentration is reduced (45). In *AOX*-overexpressing transgenic mice, presence of AOX enhanced mitochondrial respiratory rates through forward electron transport from succinate dehydrogenase (cII) both under phosphorylating (presence of ADP) and non-phosphorylating (absence of ADP) conditions (76). Lack of *AOX* in transgenic plants resulted in high ethanol production associated with injuries (118). Thus, AOX can help in decreasing fermentation and, thus can be expected to avoid harmful effects by excessively induced fermentation products (lactic acid, ethanol).

### Standard Genes Profile ‘ReprogVirus’ for Exploring Virus-Induced Early Reprogramming in Relevant Primary Infected Human Cells - A ‘Ready-to-Use’ Approach

Viruses are known to ‘abuse’ host cell’s competence and structures for reprogramming. Any virus infection provokes struggling for commanding coordination of the host cell program and this starts in the initially infected cells. Therefore, it is challenging to early stop virus-induced harmful reprogramming and avoiding at the same time suppressing the host’s defense and survival strategy. As reviewed in Costa et al. (Preprint 28), viruses typically capture host cell signaling and metabolism. Changes in host cell redox homeostasis and central carbon metabolism are recognized as most critical events during viral infection and essential for virus replication. Viruses can influence host cell cycle to arrest or progress in favor of their own replication, where E2F1 of the E2F transcription factor family plays major role. In plants, TOR-suppression by silencing or inhibition resulted in impressively reduced virus replication, resistance or elimination of viral infection. Further, host microtubule (MT) assembly is critical for virus entry, replication and spread. Enzymes catalyzing posttranslational MT modifications were identified as suitable targets for drug development to combat viral infection (119).

Based on this knowledge and the characteristics of ‘reprogramming for survival’ during SE induction and supported by our validating results on the overall approach (Preprint 28) we selected a set of genes for a ‘ready-to-use’ standard profile to explore virus-induced early reprogramming. The standard profile consists of genes related to ROS/RNS equilibration, anti-oxidant activities, NO production, G6PDH, MDH1 and 2, lactic fermentation, structural cell organization, energy status-signaling, cell cycle regulation, and regulation of apoptosis/programmed cell death and includes IRF9 and IRF3 as markers for the immune system response plus transcription factors NF-KB1 and NF-KB-RELA. The complete list of genes is given in **Table 1**.

**Table 1:** List of genes selected as ‘ReprogVirus’ for analyses in *Homo sapiens*.

Function	ReprogVirus	Gene members (accession numbers)
ROS/RNS equilibration	*ADH* (alcohol dehydrogenase)	*ADH5* (NM_000671.4)
Anti-oxidant activities	*SOD* (superoxide dismutase)	*SOD1* (NM_000454.5)
		*SOD2* (M36693.1)
	Catalase	Catalase (NM_001752.4)
	*GPX* (glutathione peroxidase)	*GPX-1* (NM_000581.4)
		*GPX-2* (NM_002083.4)
		*GPX-3* (NM_002084.5)
		*GPX-4* (NM_002085.5)
		*GPX-5* (NM_001509.3)
		*GPX-6* (NM_182701.1)
		*GPX-7* (NM_015696.5)
		*GPX-8* (NM_001008397.4)
	*GSR* (glutathione reductase)	*GSR* (NM_000637.5)
NO production	*NOS* (nitric oxide synthase)	*NOS1* (NM_000620.5),
		*NOS2* (NM_000625.4)
		*NOS3* (NM_000603.5)
Lactic fermentation	*LDH* (lactate dehydrogenase)	*LDH-A* (NM_005566.4)
		*LDH-B* (NM_002300.8)
		*LDH-C* (NM_002301.4)
		*LDH-AL6A* (NM_144972.5)
		*LDH-AL6B* (NM_033195.3)
Structural cell organization	*ACT* (Actin)	*ACT-A1* (NM_001100.4)
		*ACT-B* (NM_001101.5)
		*ACT-G1* (NM_001199954.2)
	*TUB* (Tubulin)	*TUB-A1B* (NM_006082.3)
		*TUB-A1C* (NM_001303114.1)
		*TUB-A4A* (NM_006000.3)
Glycolysis	*ENO* (Enolase)	*Eno1* (NM_001428.5)
		*Eno2* (NM_001975.3)
		*Eno3* (NM_001976.5)
	*HK* (Hexokinase)	*HK1* (NM_000188.3)
		*HK2* (NM_000189.5)
		*HK3* (NM_002115.3)
	*PFK-M* (Phosphofructokinase)	*PFK-M* (NM_001166686.2)
	*GAPDH* (Glyceraldehyde-3-phosphate dehydrogenase)	*GAPDH* (NM_002046.7)
	*PK* (Pyruvate kinase)	*PKLR* (XM_006711386.4)
		*PKM* (NM_002654.6)
Energy status-signaling	*SNRK* (sucrose non-fermenting-1-related kinase)	*SNRK* (NM_017719.5)
Cell cycle regulation	*mTOR* (target of rapamycin)	*mTOR* (NM_004958.4)
	*E2F transcription factor*	*E2F1* (NM_005225.3)
Regulation of apoptosis/cell death	*CASP* (Caspase)	Caspase in [*CASP8* (NM_001228.4); *CASP9* (NM_001229.5); *CASP10* (NM_032977.4)]
		Caspase ex [*CASP3* (NM_004346.4); *CASP6* (NM_001226.4); *CASP7* (NM_001227.5)]
	*Bcl gene*	*BCL-xL* (Z23115.1)
Markers for the immune system response	*IRF* (interferon regulatory factor)	*IRF9* (NM_006084.5), *IRF3* (NM_001571.6),
Viruses-activated transcription factors	*NF-KB1*	*NF-KB1* (NM_003998.4)
	*NF-KB-RELA*	*NF-KB-RELA* (NM_021975.4)
Other key genes	*G6PDH* (*Glucose-6-phosphate dehydrogenase)*	*G6PDH* (NM_000402.4)
	*MDH* (Malate dehydrogenase)	*MDH1* (NM_005917.4)
		*MDH2* (NM_005918.4)

## Outlook

Recent advancements in virus research increasingly reveal good relevance of transcriptome data for cell and organism performance (120–123). It is also understood that it will be important to focus on gene sets (Preprint 124). The presented standard profile of selected genes is now available to be broadly applied. It can identify critical early traits of harmful virus-induced cell reprogramming by rapid *in vitro* - screening of a diversity of virus types and variants. It should be applied under commonly accepted standard conditions in relevant human cells or tissues of primary importance for defined diseases. Currently, the profile ‘ReprogVirus’ was used by our team to trace corona virus-related reprogramming (Preprint 28). Transcriptome profiles were explored by using the data available in public domain from transcriptomic experimental studies in Genbank (NCBI). It proved to be helpful in identifying a complex SARS-CoV-2-induced trait named ‘CoV-MAC-TED’ (Preprint 28), which covers early ROS/RNS balancing, aerobic fermentation regulation and cell cycle control. Potential impact from this trait is promising to support running and new initiatives of anti-SARS-CoV-2 therapy designs as broadly discussed (Preprint 28).

**Figure 1:**
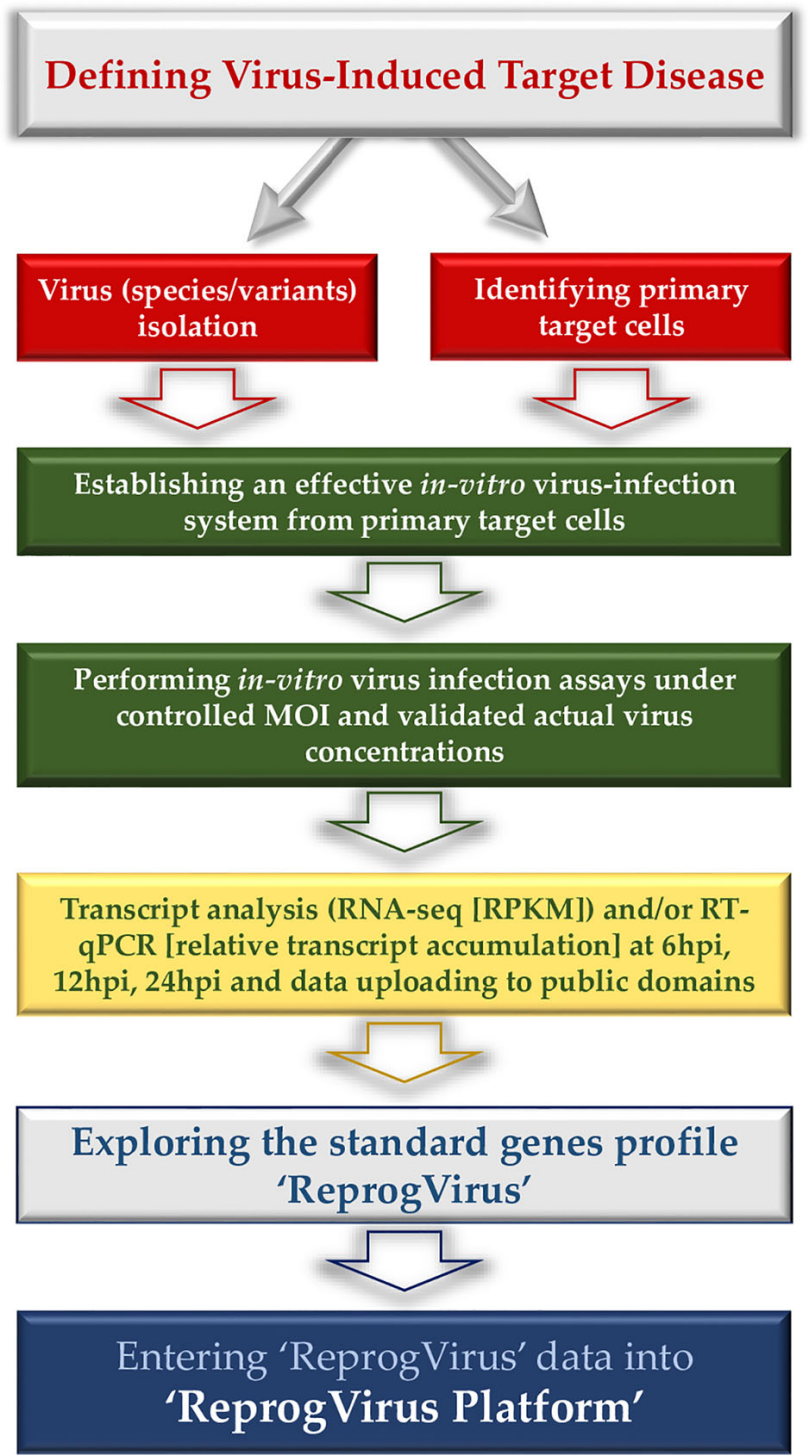
Flow diagram - data collection for ‘ReprogVirus Platform’.

Here, we announce the initiation of the ‘ReprogVirus Platform’ to enable appropriate wide data collection under standardized conditions and data processing. The strategic flow diagram in **Figure 1** provides a straightforward instruction for data collection. In parallel, regulatory data of ‘ReprogVirus’ at DNA/RNA and protein levels can be explored and collected. In case of choosing to analyze expression of individual genes (RT-qPCR), regulatory data regarding transcriptome could be obtained by exploring public databases.

The platform will provide integrative data analyses using Artificial Intelligence methodologies to identify final targets for designing specific and/or unspecific anti-viral strategies. More specifically, we intend to apply deep learning techniques to identify gene expression patterns from individual genes or from a combination of genes. These patterns will be automatically correlated with a virus or a set of viruses using a distinct deep neural network. As deep learning architecture we foresee the use of multi-head attention mechanisms in a transformer-based, variational auto-encoder network, allowing the identification of the most relevant parts of the input. Moreover, we will also apply and evaluate other CDNN (clustering deep neural networks), such as deep embedding clustering and GANs (Generative Adversarial Networks) (125).

## Data Availability Statement

The raw data supporting the conclusions of this article will be made available by the authors, without undue reservation.

## Author Contributions

BA-S initiated scientific approach and concepts in close collaboration with JHC and CN, coordinated their final development for the presented perspective through common discussions among all *FunCROP* net members and wrote the manuscript. RB contributed to manuscript writing and prepared overall ms for submission. PQ supports this initiative through his competence in Artificial Intelligence methodologies. SRK helped BA-S in overall *FunCROP* group coordination. KJG helped in writing manuscript parts related to NO metabolism. All authors contributed to the article and approved the submitted version.

## Funding

BA-S, GM, and RS acknowledge support for academic cooperation and researchers mobility by the India-Portugal Bilateral Cooperation Program (2013– 2015), funded by “Fundação para a Ciência e Tecnologia” (FCT), Portugal, and the Department of Science and Technology (DST), India. GM is grateful to UGC, India, for doctoral grant from BSR fellowship. JHC is grateful to CNPq for the Researcher fellowship (CNPq grant 309795/2017-6). KTL is grateful to CNPq for the Doctoral fellowship. SA is grateful to CAPES for the Doctoral fellowship. KJG, MO and BA-S acknowledge support by the India-Portugal Bilateral Cooperation Program ‘DST/INT/Portugal/P-03/2017’. MO Research is partially supported by National Funds through. FCT.Fundação para a Ciência e a Tecnologia, projects UIDB/04674/2020 (CIMA). BR and SS acknowledge stay support provided by DBT-TDNBC-DEAKIN – Research Network Across continents for learning and innovation (DTD-RNA) at The Energy and Resources Institute (TERI), India. BAS wants to thank Dr. Natascha Sommer for helpful discussions and comments on part of the manuscript during its development on the background of her experience as medical doctor in the group of Prof. Dr. Norbert Weissmann, Chair for ‘Molecular Mechanisms of Emphysema, Hypoxia and Lung Aging’ at the Universities of Giessen and Marburg Lung Center (UGMLC), Germany, and as investigator involved in mitochondrial redox biology also by help of transgenic AOX-mice. BA-S recognizes internal forum discussions at the University of Évora, Portugal, that helped stimulating the integration of research on viruses into our running plant research approach on cell reprogramming. CN acknowledges the international scientific network BIOALICYTED, which contributed to establish FunCROP contacts.

## Conflict of Interest

The authors declare that the research was conducted in the absence of any commercial or financial relationships that could be construed as a potential conflict of interest.

## References

[1] Haney CH Ausubel FM Urbach JM. Innate Immunity in Plants and Animals: Differences and Similarities. Biochem (Lond) (2014) 36(5):40–5. 10.1042/BIO03605040

[2] Nejat N Mantri N. Plant Immune System: Crosstalk Between Responses to Biotic and Abiotic Stresses the Missing Link in Understanding Plant Defence. Curr Issues Mol Biol (2017) 23:1–16. 10.21775/cimb.023.001 28154243

[3] Gourbal B Pinaud S Beckers GJM Van Der Meer JWM Conrath U Netea MG. Innate Immune Memory: An Evolutionary Perspective. Immunol Rev (2018) 283(1):21–40. 10.1111/imr.12647 29664574

[4] Kirman JR Quinn KM Seder RA. Immunological Memory. Immunol Cell Biol (2019) 97(7):615–6. 10.1111/imcb.12280 31283852

[5] Priyadarshini S Aich P. Effects of Psychological Stress on Innate Immunity and Metabolism in Humans: A Systematic Analysis. PLoS One (2012) 7(9):e43232. 10.1371/journal.pone.0043232 23028447 PMC3446986

[6] Delmastro-Greenwood MM Piganelli JD. Changing the Energy of an Immune Response. Am J Clin Exp Immunol (2013) 2(1):30–54. 23885324 PMC3714201

[7] Ganeshan K Nikkanen J Man K Leong YA Sogawa Y Maschek JA. Energetic Trade-Offs and Hypometabolic States Promote Disease Tolerance. Cell (2019) 177(2):399–413.e12. 10.1016/j.cell.2019.01.050 30853215 PMC6456449

[8] O’Sullivan D. The Metabolic Spectrum of Memory T Cells. Immunol Cell Biol (2019) 97(7):636–46. 10.1111/imcb.12274 31127964

[9] Steward FC Mapes MO Mears K. Growth and Organized Development of Cultured Cells. I. Growth and Division of Freely Suspended Cells. Am J Bot (1958) . 45:693–703. 10.1002/j.1537-2197.1958.tb12224.x

[10] Reinert J. Morphogenese und ihre Kontrolle an Gewebekulturen aus Karotten. Naturwissenschaften (1958) 45:344–5. 10.1007/BF00640240

[11] Grieb B Groß U Pleschka E Arnholdt-Schmitt B Neumann KH. Embryogenesis of Photoautotrophic Cell Cultures of Daucus carota L. Plant Cell Tiss Organ Cult (1994) 38:115–22. 10.1007/BF00033868

[12] Grieb B Schäfer F Imani J Mashayekhi KN Arnholdt-Schmitt B Neumann KH. Changes in Soluble Proteins and Phytohormone Concentrations of Cultured Carrot Petiole Explants During Induction of Somatic Embryogenesis (Daucus carota L.). J Appl Bot (1997) 71:94–103.

[13] Fehér A Pasternak TP Dudits D. Transition of Somatic Plant Cells to an Embryogenic State. Plant Cell Tissue Organ Cult (2003) 74:201–28. 10.1023/A:1024033216561

[14] Zavattieri MA Frederico AM Lima M Sabino R Arnholdt-Schmitt B. Induction of Somatic Embryogenesis as an Example of Stress-Related Plant Reactions. J Biotechnol (2010) 13:1. 10.2225/vol13-issue1-fulltext-4

[15] Teixeira da Silva JA Malabadi RB. Factors Affecting Somatic Embryogenesis in Conifers. J Forestry Res (2012) 23:503–15. 10.1007/s11676-012-0266-0

[16] Moon H Lee H Paek K Park S. Osmotic Stress and Strong 2,4-D Shock Stimulate Somatic-to-Embryogenic Transition in Kalopanax septemlobus (Thunb.) Koidz. Acta Physiol Plant (2015) 37:1710. 10.1007/s11738-014-1710-x

[17] Arnholdt-Schmitt B Ragonezi C Cardoso H. “A Central Role of Mitochondria for Stress-Induced Somatic Embryogenesis,” In: Germanà MA Lambardi M, editors. In Vitro Embryogenesis in Higher Plants. New York, NY: Humana Press (2016) p. 87–100. 10.1007/978-1-4939-3061-6_4 26619859

[18] Kudełko K Gaj MD. Glutathione (GSH) Induces Embryogenic Response in In Vitro Cultured Explants of Arabidopsis Thaliana Via Auxin-Related Mechanism. Plant Growth Regul (2019) 89:25–36. 10.1007/s10725-019-00514-1

[19] Smertenko A Bozhkov PV. Somatic Embryogenesis: Life and Death Processes During Apical-Basal Patterning. J Exp Bot (2014) 65(5):1343–60. 10.1093/jxb/eru005 24622953

[20] Germanà MA Lambardi M. In Vitro Embryogenesis in Higher Plants. Business Media New York: Springer Publishers (2016).

[21] Haberlandt G. Kulturversuche mit Isolierten Pflanzenzellen, Sitzungsberg. Kais. Akad Wiss Wien Mat-Naturwiss. KI Abt (1902) 111:69–92.

[22] Laimer M Rücker W. Plant Tissue Culture: 100 Years Since Gottlieb Haberlandt. Wien: Springer-Verlag Wien (2003).

[23] El-Abhar MA El-Kady MAS Ghanem KM Bosila HA. Elimination of Alfalfa Mosaic Virus (AMV) From Infected Potato Leaves (Solanum tuberosum. cv. Ditta by Embryonic Calli. J Virol Sci (2017) 1:100–13.

[24] Frederico AM Campos MD Cardoso HG Imani J Arnholdt-Schmitt B. Alternative Oxidase Involvement in Daucus carota Somatic Embryogenesis. Physiol Plant (2009) 137(4):498–508. 10.1111/j.1399-3054.2009.01278.x 19863756

[25] Cardoso HG Arnholdt-Schmitt B. “Functional Marker Development Across Species in Selected Traits”. In: Lübberstedt T Varshney RK, editors. Diagnostics in Plant Breeding. Springer Netherlands (2013). p. 467–515. 10.1007/978-94-007-5687-8_21

[26] Mohanapriya G Bharadwaj R Noceda C Costa JH Kumar SR Sathishkumar R. Alternative Oxidase (AOX) Senses Stress Levels to Coordinate Auxin-Induced Reprogramming From Seed Germination to Somatic Embryogenesis—A Role Relevant for Seed Vigor Prediction and Plant Robustness. Front Plant Sci (2019) 10:1134. 10.3389/fpls.2019.01134 31611888 PMC6776121

[27] Arnholdt-Schmitt B Costa JH de Melo DF. AOX - A Functional Marker for Efficient Cell Reprogramming Under Stress? Trends Plant Sci (2006) 11(6):281–7. 10.1016/j.tplants.2006.05.001 16713324

[28] Costa JH Mohanapriya G Bharadwaj R Noceda C Thiers KLL Shahid A. ROS/RNS Balancing, Aerobic Fermentation Regulation and Cell Cycle Control a Complex Early Trait ('CoV-MAC-TED') for Combating SARS-CoV-2-Induced Cell Reprogramming. bioRxiv (2021) 2021.06.08.447491. 10.1101/2021.06.08.447491 PMC8293103 34305903

[29] Bharadwaj R Noceda C Mohanapriya G Sathishkumar R Rajeev Kumar S Thiers KL. Adaptive Reprogramming During Early Seed Germination Requires Temporarily Enhanced Fermentation – A Critical Role for Alternative Oxidase (AOX) Regulation That Concerns Also Microbiota Effectiveness. bioRxiv (2021) 2021.06.08.447570. 10.1101/2021.06.08.447570 PMC8518632 34659277

[30] Bailey-Serres J Pierik R Ruban A Wingler A. The Dynamic Plant: Capture, Transformation, and Management of Energy. Plant Physiol (2018) 176(2):961–6. 10.1104/pp.18.00041 PMC5813544 29438068

[31] Schmidt RR Weits DA Feulner CFJ van Dongen JT. Oxygen Sensing and Integrative Stress Signaling in Plants. Plant Physiol (2018) 176(2):1131–42. 10.1104/pp.17.01394 PMC5813526 29162635

[32] Sakr S Wang M Dédaldéchamp F Perez-Garcia MD Ogé L Hamama L. The Sugar-Signaling Hub: Overview of Regulators and Interaction With the Hormonal and Metabolic Network. Int J Mol Sci (2018) 19(9):2506. 10.3390/ijms19092506 PMC6165531 30149541

[33] Wurzinger B Nukarinen E Nägele T Weckwerth W Teige M. The SnRK1 Kinase as Central Mediator of Energy Signaling Between Different Organelles. Plant Physiol (2018) 176(2):1085–94. 10.1104/pp.17.01404 PMC5813556 29311271

[34] Wang WR Liang JH Wang GF Sun MX Peng FT Xiao YS. Overexpression of Ppsnrk1α in Tomato Enhanced Salt Tolerance by Regulating ABA Signaling Pathway and Reactive Oxygen Metabolism. BMC Plant Biol (2020) 20(1):128. 10.1186/s12870-020-02342-2 32216751 PMC7099830

[35] Sangüesa G Roglans N Baena M Velázquez AM Laguna JC Alegret M. mTOR is a Key Protein Involved in the Metabolic Effects of Simple Sugars. Int J Mol Sci (2019) 20(5):1117. 10.3390/ijms20051117 PMC6429387 30841536

[36] Ahmad Z Magyar Z Bögre L Papdi C. Cell Cycle Control by the Target of Rapamycin Signalling Pathway in Plants. J Exp Bot (2019) 70(8):2275–84. 10.1093/jxb/erz140 30918972

[37] Ryabova LA Robaglia C Meyer C. Target of Rapamycin Kinase: Central Regulatory Hub for Plant Growth and Metabolism. J Exp Bot (2019) 70(8):2211–6. 10.1093/jxb/erz108 PMC6463030 30984977

[38] Baena-González E Hanson J. Shaping Plant Development Through the SnRK1-TOR Metabolic Regulators. Curr Opin Plant Biol (2017) 35:152–7. 10.1016/j.pbi.2016.12.004 28027512

[39] Mellema S Eichenberger W Rawyler A Suter M Tadege M Kuhlemeier C. The Ethanolic Fermentation Pathway Supports Respiration and Lipid Biosynthesis in Tobacco Pollen. Plant J (2002) 30(3):329–36. 10.1046/j.1365-313x.2002.01293.x 12000680

[40] Pasternak T Dudits D. Epigenetic Clues to Better Understanding of the Asexual Embryogenesis in Planta and In Vitro. Front Plant Sci (2019) 10:778. 10.3389/fpls.2019.00778 31275336 PMC6592144

[41] Perata P Loschiavo F Alpi A. Ethanol Production and Toxicity in Suspension Cultured Carrot Cells and Embryos. Planta (1988) 173(3):322–9. 10.1007/BF00401019 24226539

[42] Perata P Pozueta-Romero J Akazawa T Yamaguchi J. Effect of Anoxia on Starch Breakdown in Rice and Wheat Seeds. Planta (1992) 188(4):611–8. 10.1007/BF00197056 24178396

[43] Fan Y Yu X Guo H Wei J Guo H Zhang L. Dynamic Transcriptome Analysis Reveals Uncharacterized Complex Regulatory Pathway Underlying Dose Iba Induced Embryogenic Redifferentiation in Cotton. Int J Mol Sci (2020) 21(2):426. 10.3390/ijms21020426 PMC7013799 31936561

[44] Nguyen KH Mostofa MG Watanabe Y Tran CD Rahman M Tran LP. Overexpression of GmNAC085 Enhances Drought Tolerance in Arabidopsis by Regulating Glutathione Biosynthesis, Redox Balance and Glutathione-Dependent Detoxification of Reactive Oxygen Species and Methylglyoxal. Environ Exp Bot (2019) 161:242–54. 10.1016/j.envexpbot.2018.12.021

[45] Zabalza A van Dongen JT Froehlich A Oliver SN Faix B Gupta KJ. Regulation of Respiration and Fermentation to Control the Plant Internal Oxygen Concentration. Plant Physiol (2009) 149(2):1087–98. 10.1104/pp.108.129288 PMC2633817 19098094

[46] Fehér A. Somatic Embryogenesis - Stress-induced Remodeling of Plant Cell Fate. Biochim Biophys Acta (2015) 1849(4):385–402. 10.1016/j.bbagrm.2014.07.005 25038583

[47] Hazubska-Przybył T Ratajczak E Obarska A Pers-Kamczyc E. Different Roles of Auxins in Somatic Embryogenesis Efficiency in Two Picea Species. Int J Mol Sci (2020) 21(9):3394. 10.3390/ijms21093394 PMC7246981 32403374

[48] Zorov DB Filburn CR Klotz LO Zweier JL Sollott SJ. Reactive Oxygen Species (ROS)-Induced ROS Release: A New Phenomenon Accompanying Induction of the Mitochondrial Permeability Transition in Cardiac Myocytes. J Exp Med (2000). 192(7):1001–14. 10.1084/jem.192.7.1001 PMC2193314 11015441

[49] Bigarella CL Liang R Ghaffari S. Stem Cells and the Impact of ROS Signaling. Development (2014) 141(22):4206–18. 10.1242/dev.107086 PMC4302918 25371358

[50] Gupta KJ Kolbert Z Durner J Lindermayr C Corpas FJ Brouquisse R. Regulating the Regulator: Nitric Oxide Control of Post-Translational Modifications. New Phytol (2020) 227(5):1319–25. 10.1111/nph.16622 32339293

[51] Zamoraa R Bryan NS Boyle P Wong C Milsom BA Jaffe R. Nitrosative Stress in an Animal Model of Necrotizing Enterocolitis. Free Radical Biol Med (2005) 39(11):1428–37. 10.1016/j.freeradbiomed.2005.07.004 16274878

[52] Dumont S Rivoal J. Consequences of Oxidative Stress on Plant Glycolytic and Respiratory Metabolism. Front Plant Sci (2019) 10:166. 10.3389/fpls.2019.00166 30833954 PMC6387960

[53] Qi W Ma L Wang F Wang P Wu J Jin J. Reactive Oxygen Species as Important Regulators of Cell Division. bioRxiv (2020). 10.1101/2020.03.06.980474

[54] Pengpeng J Chenyu D Penghu C Dong S Ruizhuo O Yuqing M. The Role of Reactive Oxygen Species in Tumor Treatment. RSC Adv (2020) 10(13):7740–50. 10.1039/C9RA10539E PMC9049915 35492191

[55] Gupta KJ Hancock JT Petrivalsky M Kolbert Z Lindermayr C Durner J. Recommendations on Terminology and Experimental Best Practice Associated With Plant Nitric Oxide Research. New Phytol (2020) 225(5):1828–34. 10.1111/nph.16157 31479520

[56] Bui LT Novi G Lombardi L Iannuzzi C Rossi J Santaniello A. Conservation of Ethanol Fermentation and Its Regulation in Land Plants. J Exp Bot (2019) 70(6):1815–27. 10.1093/jxb/erz052 PMC6436157 30861072

[57] Shi YF Wang DL Wang C Culler AH Kreiser MA Suresh J. Loss of GSNOR1 Function Leads to Compromised Auxin Signaling and Polar Auxin Transport. Mol Plant (2015) 8(9):1350–65. 10.1016/j.molp.2015.04.008 25917173

[58] Rizza S Filomeni G. Role, Targets and Regulation of (De)Nitrosylation in Malignancy. Front Oncol (2018) 8:334. 10.3389/fonc.2018.00334 30234010 PMC6131587

[59] Barnett SD Buxton ILO. The Role of S-Nitrosoglutathione Reductase (GSNOR) in Human Disease and Therapy. Crit Rev Biochem Mol Biol (2017) 52(3):340–54. 10.1080/10409238.2017.1304353 PMC5597050 28393572

[60] Gong B Wen D Wang X Wei M Yang F Li Y. S-Nitrosoglutathione Reductase-Modulated Redox Signaling Controls Sodic Alkaline Stress Responses in Solanum lycopersicum L. Plant Cell Physiol (2015) 56(4):790–802. 10.1093/pcp/pcv007 25634962

[61] Anthony J Senaratna T Dixon K Sivasithamparam K. The Role of Antioxidants for Initiation of Somatic Embryos With Conostephium pendulum (Ericaceae). Plant Cell Tissue Organ Cult (2004) 78:247–52. 10.1023/B:TICU.0000025661.56250.b4

[62] Jariteh M Ebrahimzadeh H Niknam V Mirmasoumi M Vahdati K. Developmental Changes in Protein, Proline and Some Antioxidant Enzymes Activities in Somatic and Zygotic Embryos of Persian Walnut (Juglans regia L.). Plant Cell Tissue Organ Cult (2015) 122:101–15. 10.1007/s11240-015-0753-z

[63] Gomez-Garay A Lopez JA Camafeita E Bueno MA Pintos B. Proteomic Perspective of Quercus Suber Somatic Embryogenesis. J Proteomics (2013) 93:314–25. 10.1016/j.jprot.2013.06.006 23770300

[64] Reis E Batista MT Canhoto JM. Effect and Analysis of Phenolic Compounds During Somatic Embryogenesis Induction in Feijoa Sellowiana Berg. Protoplasma (2008) 232(3-4):193–202. 10.1007/s00709-008-0290-2 18767216

[65] Bahmankar M Mortazavian SMM Tohidfar M Sadat Noori SA Izadi Darbandi A Corrado G. Chemical Compositions, Somatic Embryogenesis, and Somaclonal Variation in Cumin. BioMed Res Int (2017) 2017:7283806. 10.1155/2017/7283806 29234682 PMC5694991

[66] Mira MM Hill RD Stasolla C. Phytoglobins Improve Hypoxic Root Growth by Alleviating Apical Meristem Cell Death. Plant Physiol (2016) 172(3):2044–56. 10.1104/pp.16.01150 PMC5100795 27702845

[67] Elhiti M Huang S Mira MM Hill RD Stasolla C. Redirecting Cell Fate During In Vitro Embryogenesis: Phytoglobins as Molecular Switches. Front Plant Sci (2018) 9:1477. 10.3389/fpls.2018.01477 30356752 PMC6189464

[68] Vishwakarma A Kumari A Mur LAJ Gupta KJ. A Discrete Role for Alternative Oxidase Under Hypoxia to Increase Nitric Oxide and Drive Energy Production. Free Radic Biol Med (2018) 122:40–51. 10.1016/j.freeradbiomed.2018.03.045 29604396

[69] McDonald AE. Alternative Oxidase: An Inter-Kingdom Perspective on the Function and Regulation of This Broadly Distributed ‘Cyanide-Resistant’ Terminal Oxidase. Funct Plant Biol (2008) 35(7):535–52. 10.1071/FP08025 32688810

[70] McDonald AE Amirsadeghi S Vanlerberghe GC. Prokaryotic Orthologues of Mitochondrial Alternative Oxidase and Plastid Terminal Oxidase. Plant Mol Biol (2003) 53(6):865–76. 10.1023/B:PLAN.0000023669.79465.d2 15082931

[71] Atteia A van Lis R van Hellemond JJ Tielens AG Martin W Henze K. Identification of Prokaryotic Homologues Indicates an Endosymbiotic Origin for the Alternative Oxidases of Mitochondria (AOX) and Chloroplasts (PTOX). Gene (2004) 330:143–8. 10.1016/j.gene.2004.01.015 15087133

[72] Vicente C Costa JH Arnholdt-Schmitt B. “Bacterial AOX: A Provocative Lack of Interest!” In: Gupta KJ Mur LA Neelwarne B, editors. Alternative Respiratory Pathways in Higher Plants. Oxford: Wiley Publishing group (2015). p. 319–22. 10.1002/9781118789971.ch23

[73] Rustin P Jacobs HT. Respiratory Chain Alternative Enzymes as Tools to Better Understand and Counteract Respiratory Chain Deficiencies in Human Cells and Animals. Physiol Plant (2009) 137(4):362–70. 10.1111/j.1399-3054.2009.01249.x 19508504

[74] Dassa EP Dufour E Goncalves S Jacobs HT Rustin P. The Alternative Oxidase, a Tool for Compensating Cytochrome C Oxidase Deficiency in Human Cells. Physiol Plant (2009) 137(4):427–34. 10.1111/j.1399-3054.2009.01248.x 19493305

[75] Szibor M Dhandapani PK Dufour E Holmström KM Zhuang Y Salwig I. Broad AOX Expression in a Genetically Tractable Mouse Model Does Not Disturb Normal Physiology. Dis Model Mech (2017) 10(2):163–71. 10.1242/dmm.027839 PMC5312010 28067626

[76] Szibor M Gainutdinov T Fernandez-Vizarra E Dufour E Gizatullina Z Debska-Vielhaber G. Bioenergetic Consequences From Xenotopic Expression of a Tunicate AOX in Mouse Mitochondria: Switch From RET and ROS to FET. Biochim Biophys Acta Bioenerg (2020) 1861(2):148137. 10.1016/j.bbabio.2019.148137 31825809

[77] Rajendran J Purhonen J Tegelberg S Smolander OP Mörgelin M Rozman J. Alternative Oxidase-Mediated Respiration Prevents Lethal Mitochondrial Cardiomyopathy. EMBO Mol Med (2019) 11(1):e9456. 10.15252/emmm.201809456 30530468 PMC6328925

[78] Kemppainen KK Kemppainen E Jacobs HT. The Alternative Oxidase AOX Does Not Rescue the Phenotype of tko25t Mutant Flies. G3 (Bethesda) (2014) 4(10):2013–21. 10.1534/g3.114.013946 PMC4199707 25147191

[79] Sommer N Alebrahimdehkordi N Pak O Knoepp F Strielkov I Scheibe S. Bypassing Mitochondrial Complex III Using Alternative Oxidase Inhibits Acute Pulmonary Oxygen Sensing. Sci Adv (2020) 6(16):eaba0694. 10.1126/sciadv.aba0694 32426457 PMC7159913

[80] Selemidis S. Targeting Reactive Oxygen Species for Respiratory Infection: Fact or Fancy? Respirology (2019) 24(1):15–6. 10.1111/resp.13417 30295360

[81] To EE Erlich JR Liong F Luong R Liong S Esaq F. Mitochondrial Reactive Oxygen Species Contribute to Pathological Inflammation During Influenza A Virus Infection in Mice. Antioxid Redox Signal (2020) 32(13):929–42. 10.1089/ars.2019.7727 PMC7104903 31190565

[82] El-Khoury R Dufour E Rak M Ramanantsoa N Grandchamp N Csaba Z. Alternative Oxidase Expression in the Mouse Enables Bypassing Cytochrome C Oxidase Blockade and Limits Mitochondrial ROS Overproduction. PLoS Genet (2013) 9(1):e1003182. 10.1371/journal.pgen.1003182 23300486 PMC3536694

[83] Giordano L Farnham A Dhandapani PK Salminen L Bhaskaran J Voswinckel R. Alternative Oxidase Attenuates Cigarette Smoke-Induced Lung Dysfunction and Tissue Damage. Am J Respir Cell Mol Biol (2019) 60(5):515–22. 10.1165/rcmb.2018-0261OC PMC6503618 30339461

[84] Lee Hansen D Church JN Matheson S McCarlie VW Thygerson T Criddle RS. Kinetics of Plant Growth and Metabolism. Thermochimica Acta (2002) 388(1–2):415–25. 10.1016/S0040-6031(02)00021-7

[85] Clifton R Millar AH Whelan J. Alternative Oxidases in Arabidopsis: A Comparative Analysis of Differential Expression in the Gene Family Provides New Insights Into Function of non-Phosphorylating Bypasses. Biochim Biophys Acta (2006) 1757(7):730–41. 10.1016/j.bbabio.2006.03.009 16859634

[86] Rasmusson AG Fernie AR van Dongen JT. Alternative Oxidase: A Defence Against Metabolic Fluctuations? Physiol Plant (2009) 137(4):371–82. 10.1111/j.1399-3054.2009.01252.x 19558416

[87] Vanlerberghe GC Cvetkovska M Wang J. Is the Maintenance of Homeostatic Mitochondrial Signaling During Stress a Physiological Role for Alternative Oxidase? Physiol Plant (2009) 137(4):392–406. 10.1111/j.1399-3054.2009.01254.x 19549065

[88] Zhang L Oh Y Li H Baldwin IT Galis I. Alternative Oxidase in Resistance to Biotic Stresses: Nicotiana Attenuata AOX Contributes to Resistance to a Pathogen and a Piercing-Sucking Insect But Not Manduca Sexta Larvae. Plant Physiol (2012) 160(3):1453–67. 10.1104/pp.112.200865 PMC3490609 22961128

[89] Vanlerberghe GC. Alternative Oxidase: A Mitochondrial Respiratory Pathway to Maintain Metabolic and Signaling Homeostasis During Abiotic and Biotic Stress in Plants. Int J Mol Sci (2013) 14(4):6805–47. 10.3390/ijms14046805 PMC3645666 23531539

[90] Rogov AG Sukhanova EI Uralskaya LA Aliverdieva DA Zvyagilskaya RA. Alternative Oxidase: Distribution, Induction, Properties, Structure, Regulation, and Functions. Biochem (Mosc) (2014) 79(13):1615–34. 10.1134/S0006297914130112 25749168

[91] Selinski J Scheibe R Day DA Whelan J. Alternative Oxidase Is Positive for Plant Performance. Trends Plant Sci (2018) 23(7):588–97. 10.1016/j.tplants.2018.03.012 29665989

[92] Murphy AM Zhou T Carr JP. An Update on Salicylic Acid Biosynthesis, its Induction and Potential Exploitation by Plant Viruses. Curr Opin Virol (2020) 42:8–17. 10.1016/j.coviro.2020.02.008 32330862

[93] Carr JP Murphy AM Tungadi T Yoon JY. Plant Defense Signals: Players and Pawns in Plant-Virus-Vector Interactions. Plant Sci (2019) 279:87–95. 10.1016/j.plantsci.2018.04.011 30709497

[94] Fu LJ Shi K Gu M Zhou YH Dong DK Liang WS. Systemic Induction and Role of Mitochondrial Alternative Oxidase and Nitric Oxide in a Compatible Tomato- Tobacco Mosaic Virus Interaction. Mol Plant Microbe Interact (2010) 23(1):39–48. 10.1094/MPMI-23-1-0039 19958137

[95] Santos Macedo E Cardoso HG Hernández A Peixe AA Polidoros A Ferreira A. Physiologic Responses and Gene Diversity Indicate Olive Alternative Oxidase as a Potential Source for Markers Involved in Efficient Adventitious Root Induction. Physiol Plant (2009) 137(4):532–52. 10.1111/j.1399-3054.2009.01302.x 19941624

[96] Velada I Grzebelus D Lousa D M Soares C Santos Macedo E Peixe A. AOX1-Subfamily Gene Members in Olea europaea cv. “Galega Vulgar”-Gene Characterization and Expression of Transcripts During IBA Induced In Vitro Adventitious Rooting. Int J Mol Sci (2018) 17;19(2):597. 10.3390/ijms19020597 PMC5855819 29462998

[97] Campos MD Cardoso HG Linke B Costa JH de Melo DF Justo L. Differential Expression and Co-Regulation of Carrot AOX Genes (Daucus carota). Physiol Plant (2009) 137(4):578–91. 10.1111/j.1399-3054.2009.01282.x 19825008

[98] Campos MD Nogales A Cardoso HG Kumar SR Nobre T Sathishkumar R. Stress-Induced Accumulation of DcAOX1 and DcAOX2a Transcripts Coincides With Critical Time Point for Structural Biomass Prediction in Carrot Primary Cultures (Daucus carota L.). Front Genet (2016) 7:1. 10.3389/fgene.2016.00001 26858746 PMC4731517

[99] Velada I Cardoso HG Ragonezi C Nogales A Ferreira A Valadas V. Alternative Oxidase Gene Family in Hypericum perforatum L.: Characterization and Expression at the Post-Germinative Phase. Front Plant Sci (2016) 7:1043. 10.3389/fpls.2016.01043 27563303 PMC4980395

[100] Ivanova A Law SR Narsai R Duncan O Lee JH Zhang B. A Functional Antagonistic Relationship Between Auxin and Mitochondrial Retrograde Signaling Regulates Alternative Oxidase1a Expression in Arabidopsis. Plant Physiol (2014) 165(3):1233–54. 10.1104/pp.114.237495 PMC4081334 24820025

[101] Wang Y Berkowitz O Selinski J Xu Y Hartmann A Whelan J. Stress Responsive Mitochondrial Proteins in Arabidopsis Thaliana. Free Radic Biol Med (2018) 122:28–39. 10.1016/j.freeradbiomed.2018.03.031 29555593

[102] Scheibe R. Maintaining Homeostasis by Controlled Alternatives for Energy Distribution in Plant Cells Under Changing Conditions of Supply and Demand. Photosynth Res (2019) 139(1-3):81–91. 10.1007/s11120-018-0583-z 30203365 PMC6373317

[103] Del-Saz NF Ribas-Carbo M McDonald AE Lambers H Fernie AR Florez- Sarasa I. An In Vivo Perspective of the Role(s) of the Alternative Oxidase Pathway. Trends Plant Sci (2018) 23(3):206–19. 10.1016/j.tplants.2017.11.006 29269217

[104] Selinski J Hartmann A Deckers-Hebestreit G Day DA Whelan J Scheibe R. Alternative Oxidase Isoforms Are Differentially Activated by Tricarboxylic Acid Cycle Intermediates. Plant Physiol (2018) 176(2):1423–32. 10.1104/pp.17.01331 PMC5813554 29208641

[105] Vishwakarma A Tetali SD Selinski J Scheibe R Padmasree K. Importance of the Alternative Oxidase (AOX) Pathway in Regulating Cellular Redox and ROS Homeostasis to Optimize Photosynthesis During Restriction of the Cytochrome Oxidase Pathway in Arabidopsis Thaliana. Ann Bot (2015) 116(4):555–69. 10.1093/aob/mcv122 PMC4578005 26292995

[106] Cvetkovska M Vanlerberghe GC. Alternative Oxidase Modulates Leaf Mitochondrial Concentrations of Superoxide and Nitric Oxide. New Phytol (2012) 195(1):32–9. 10.1111/j.1469-8137.2012.04166.x 22537177

[107] Cvetkovska M Dahal K Alber NA Jin C Cheung M Vanlerberghe GC. Knockdown of Mitochondrial Alternative Oxidase Induces the ‘Stress State’ of Signalling Molecule Pools in Nicotiana Tabacum, With Implications for Stomatal Function. New Phytol (2014) 203(2):449–61. 10.1111/nph.12773 24635054

[108] Szal B Lukawska K Zdolińska I Rychter AM. Chilling Stress and Mitochondrial Genome Rearrangement in the MSC16 Cucumber Mutant Affect the Alternative Oxidase and Antioxidant Defense System to a Similar Extent. Physiol Plant (2009) 137(4):435–45. 10.1111/j.1399-3054.2009.01255.x 19549067

[109] Amirsadeghi S Robson CA McDonald AE Vanlerberghe GC. Changes in Plant Mitochondrial Electron Transport Alter Cellular Levels of Reactive Oxygen Species and Susceptibility to Cell Death Signaling Molecules. Plant Cell Physiol (2006) 47(11):1509– 19. 10.1093/pcp/pcl016 17012741

[110] Amirsadeghi S Robson CA Vanlerberghe GC. The Role of the Mitochondrion in Plant Responses to Biotic Stress. Physiol Plant (2007) 129:253–66. 10.1111/j.1399-3054.2006.00775.x

[111] Hanqing F Kun S Mingquan L Hongyu L Xin L Yan L. The Expression, Function and Regulation of Mitochondrial Alternative Oxidase Under Biotic Stresses. Mol Plant Pathology (2010) 11:429–40. 10.1111/j.1364-3703.2010.00615.x PMC6640418 20447290

[112] Hernández JA Gullner G Clemente-Moreno MJ Künstler A Juhász C Díaz-Vivancos P. Oxidative Stress and Antioxidative Responses in Plant–Virus Interactions. Physiol Mol Plant Pathol (2016) . 94:134–48. 10.1016/j.pmpp.2015.09.001

[113] Millar AH Hoefnagel M Day DA Wiskich JT. Specificity of the Organic Acid Activation of Alternative Oxidase in Plant Mitochondria. Plant Physiol (1996) 111(2):613–8. 10.1104/pp.111.2.613 PMC157873 12226315

[114] Hoefnagel M Rich PR Zhang Q Wiskich JT. Substrate Kinetics of the Plant Mitochondrial Alternative Oxidase and the Effects of Pyruvate. Plant Physiol (1997) 115(3):1145–53. 10.1104/pp.115.3.1145 PMC158579 12223863

[115] Albury MS Elliott C Moore AL. Towards a Structural Elucidation of the Alternative Oxidase in Plants. Physiol Plant (2009) 137(4):316–27. 10.1111/j.1399-3054.2009.01270.x 19719482

[116] Hakkaart GA Dassa EP Jacobs HT Rustin P. Allotopic Expression of a Mitochondrial Alternative Oxidase Confers Cyanide Resistance to Human Cell Respiration. EMBO Rep (2006) 7(3):341–5. 10.1038/sj.embor.7400601 PMC1456879 16322757

[117] Carré JE Affourtit C Moore AL. Interaction of Purified Alternative Oxidase From Thermogenic Arum Maculatum With Pyruvate. FEBS Lett (2011) 585(2):397–401. 10.1016/j.febslet.2010.12.026 21187094

[118] Vanlerberghe GC Vanlerberghe AE McIntosh L. Molecular Genetic Alteration of Plant Respiration: Silencing and Overexpression of Alternative Oxidase in Transgenic Tobacco. Plant Physiol (1994) 106(4):1503–10. 10.1104/pp.106.4.1503 PMC159691 12232424

[119] Oláh J Lehotzky A Szunyogh S Szénási T Orosz F Ovádi J. Microtubule- Associated Proteins With Regulatory Functions by Day and Pathological Potency at Night. Cells (2020) 9(2):357. 10.3390/cells9020357 PMC7072251 32033023

[120] Šubr Z Predajňa L Šoltys K Bokor B Budiš J Glasa M. Comparative Transcriptome Analysis of Two Cucumber Cultivars With Different Sensitivity to Cucumber Mosaic Virus Infection. Pathogens (2020) 9(2):145. 10.3390/pathogens9020145 PMC7168641 32098056

[121] Davidson AD Williamson MK Lewis S Shoemark D Carroll MW Heesom KJ. Characterisation of the Transcriptome and Proteome of SARS-CoV-2 Reveals a Cell Passage Induced in-Frame Deletion of the Furin-Like Cleavage Site From the Spike Glycoprotein. Genome Med (2020) . 12(1):68. 10.1186/s13073-020-00763-0 32723359 PMC7386171

[122] Lima MC de Mendonça LR Rezende AM Carrera RM Aníbal-Silva CE Demers M. The Transcriptional and Protein Profile From Human Infected Neuroprogenitor Cells Is Strongly Correlated to Zika Virus Microcephaly Cytokines Phenotype Evidencing a Persistent Inflammation in the CNS. Front Immunol (2019) . 10:1928. 10.3389/fimmu.2019.01928 31474994 PMC6707094

[123] Wynne JW Todd S Boyd V Tachedjian M Klein R Shiell B. Comparative Transcriptomics Highlights the Role of the Activator Protein 1 Transcription Factor in the Host Response to Ebolavirus. J Virol (2017) 91(23):e01174–17. 10.1128/JVI.01174-17 PMC5686711 28931675

[124] Kuchi S Gu Q Palmarini M Wilson SJ Robertson DL. Meta-Analysis of Virus-Induced Host Gene Expression Reveals Unique Signatures of Immune Dysregulation Induced by SARS-Cov-2. bioRxiv (2020). 10.1101/2020.12.29.424739

[125] Karim MR Beyan O Zappa A Costa IG Rebholz-Schuhmann D Cochez M. Deep Learning-Based Clustering Approaches for Bioinformatics. Brief Bioinform (2021) 22(1):393–415. 10.1093/bib/bbz170 32008043 PMC7820885

